# Design Optimization of a Soft Robotic Rehabilitation Glove Based on Finger Workspace Analysis

**DOI:** 10.3390/biomimetics9030172

**Published:** 2024-03-13

**Authors:** Yechan Lee, Hyung-Soon Park

**Affiliations:** Department of Mechanical Engineering, Korea Advanced Institute of Science and Technology, Daejeon 34141, Republic of Korea; neo9se@kaist.ac.kr

**Keywords:** design optimization, soft robot, finger workspace, rehabilitation robot, hand rehabilitation

## Abstract

The finger workspace is crucial for performing various grasping tasks. Thus, various soft rehabilitation gloves have been developed to assist individuals with paralyzed hands in activities of daily living (ADLs) or rehabilitation training. However, most soft robotic glove designs are insufficient to assist with various hand postures because most of them use an underactuated mechanism for design simplicity. Therefore, this paper presents a methodology for optimizing the design of a high-degree-of-freedom soft robotic glove while not increasing the design complexity. We defined the required functional workspace of the index finger based on ten frequently used grasping postures in ADLs. The design optimization was achieved by simulating the proposed finger–robot model to obtain a comparable workspace to the functional workspace. In particular, the moment arm length for extension was optimized to facilitate the grasping of large objects (precision disk and power sphere), whereas a torque-amplifying routing design was implemented to aid the grasping of small objects (lateral pinch and thumb–two-finger pinch). The effectiveness of the optimized design was validated through testing with a stroke survivor and comparing the assistive workspace. The observed workspace demonstrated that the optimized glove design could assist with nine out of the ten targeted grasping posture functional workspaces. Furthermore, the assessment of the grasping speed and force highlighted the glove’s usability for various rehabilitation activities. We also present and discuss a generalized methodology to optimize the design parameters of a soft robotic glove that uses an underactuated mechanism to assist the targeted workspace. Overall, the proposed design optimization methodology serves as a tool for developing advanced hand rehabilitation robots, as it offers insight regarding the importance of routing optimization in terms of the workspace.

## 1. Introduction

The loss of fine motor skills is a prevalent issue in neurological conditions and considerably impacts the quality of life of the affected individuals [1]. In the United States, over 7 million adults have experienced a stroke; approximately half of them struggle to fully regain the hand function required for the independent performance of activities of daily living (ADLs) [2,3]. Moreover, in the United States, over 0.2 million people have a spinal cord injury (SCI) [4]. The restoration of arm and hand functions is crucial for improving patients’ quality of life with a quadriplegic SCI [5]. However, individuals with neurological diseases often encounter challenges owing to weakened finger muscles, making it difficult to grasp and manipulate objects during ADLs [6,7].

To address these issues, hand rehabilitation focuses on restoring the functions that are essential for ADLs, customized to individual needs. A key rehabilitation method is task-oriented training, which involves guiding patients through specific tasks, such as reaching and grasping. Tailoring tasks to individuals yields greater efficiency than generic tasks [8]. However, the intricate hand postures required in individualized task-oriented training, combined with the delicate nature of the hand, present notable challenges. Additionally, physical therapists bear a demanding workload that requires substantial time and effort [9]. As a result, several researchers have focused on developing robotic devices for hand rehabilitation to ease the burden on therapists and provide advanced hand assistance through devices, such as exoskeletons and soft robots [10].

Rehabilitation robots for the hand have been a hot research topic, with exoskeletons and soft robotic gloves being the prominent types. Exoskeletons, which are characterized by a rigid structure with actuators attached to the forearm or back of the hand, can provide firm support for hand postures and grasping. However, the rigid nature of exoskeletons may pose challenges with regard to comfort and natural movement, especially considering that patients typically have weakened arm strength [11,12]. In contrast, soft robotic gloves offer flexibility and adaptability, potentially providing a more comfortable and natural user experience. For instance, Gloreha employed five pneumatic cylinders located away from the hand to reduce the weight and assist with task-oriented rehabilitation therapy [13]. Another example is the Exo-Glove Poly, which features a cable-driven mechanism for actuation and adjustability to fit different hand sizes [14]. This soft robotic glove can assist patients with an SCI in flexing and extending their fingers for grasping tasks. RELab tenoexo is a lightweight wearable device that assists with four grasping postures using two actuators and one slider mechanism [15]. Two soft compliant finger mechanisms provide combined actuation of digits 2–5 and the thumb, with the thumb abduction/adduction slider being manually adjustable for different grasping postures.

Despite notable advancements, most soft robots are constrained to adopt an underactuated mechanism, facilitating only the opening and closing motions of the fingers to minimize the number of actuators for weight reduction and a compliant design on the hand. However, human fingers perform more complex roles beyond simple opening and closing, including various grasping postures. The limited motions of the simple opening and closing project the fingertip’s workspace into one curve, highlighting the need for various combinations of joint angles to enable the fingertip to reach a larger workspace.

The KAIST dexterous glove (KADEX), which is a high-degree-of-freedom (high-DoF) soft wearable robot, marks a considerable departure from conventional soft robots since it allows for assistance with various hand postures [16]. KADEX assists the five fingers with eight cables. Each finger receives assistance from at least one cable, designated as a flexion cable, thereby enabling individual assistance to each finger. Notably, an additional cable is allocated to facilitate assistance for the thumb, index finger, and middle finger, enhancing the versatility in hand movements and postures. The operating principle of KADEX resembles that of a drone, with the capability to control the altitude through upper directional propulsion only. Continuous tension from the silicone strap on the dorsal side of the finger extends the finger joints, analogous to the gravitational pull on a drone. A flexion cable on the opposite side also imparts flexion torque to all joints. Intrinsic cables for the index and middle fingers aid in flexing the metacarpophalangeal (MCP) joint and extending the proximal interphalangeal (PIP) joint. This combined tension from the silicone straps and cables allows for bidirectional control of the interjoint coordination of the MCP and PIP joints [16,17]. Moreover, it enables stroke survivors to perform various hand postures, including a medium wrap, power sphere, lateral pinch, thumb–two-finger pinch, and tripod grip, for ADLs, such as eating, writing, and using a smartphone [16]. The significant advancement in the increased DoFs of KADEX has resulted in an expanded workspace compared with other soft glove designs. However, like the other soft robots, KADEX uses an underactuated mechanism to assist with extension motion, which brings uncertainty to its workspace due to the various hand characteristics of the user.

Herein, we propose a method for optimizing the design of a high-DoF soft robotic glove to enhance its capability to assist with various grasping postures in ADLs. Initially, we defined the functional workspace of the index finger, encompassing ten frequently employed grasping postures that represent over 80% of ADL tasks [18]. To validate the glove’s ability to accommodate this functional workspace, we constructed a finger–robot model to simulate the glove workspace. Subsequently, we evaluated the robot’s capacity to assist with the functional workspace. We then determined the optimized moment arm length of the silicone strap and refined the cable routing design to expand the robot’s workspace to align with the functional workspace. Finally, we conducted experimental validation of the optimized design’s workspace for a stroke subject, juxtaposing it against the workspace of the previous KADEX design and the predefined functional workspace. Ultimately, we assessed whether the optimized design could provide an adequate workspace, speed, and force for various grasping tasks in dexterous ADLs. Moreover, we discuss and offer insights into adjusting the moment arm of the high-DoF cable-driven glove to facilitate a larger workspace.

## 2. Materials and Methods

**Figure 1:**
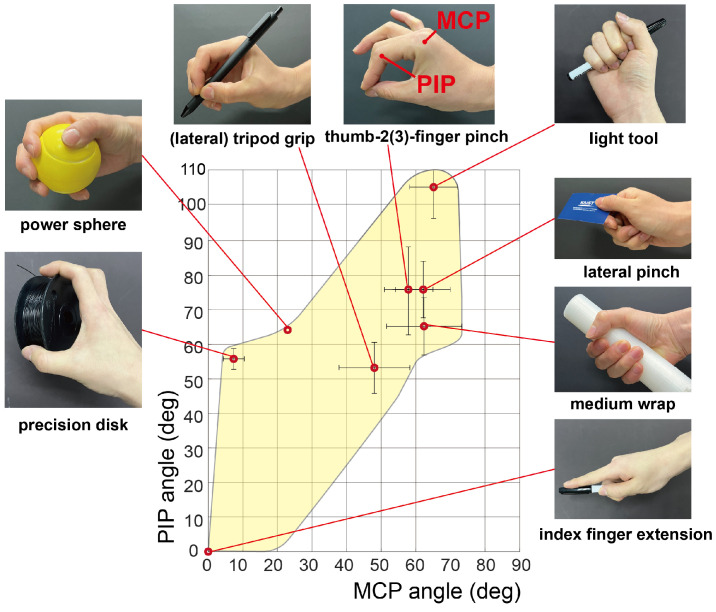
Yellow area represents the functional workspace for ten grasping postures. A set of MCP and PIP joint angles of the index finger represent each grasping posture (red dot). Error bars denote standard deviations. Certain postures share identical joint angles at the index finger, such as thumb–2-finger pinch and thumb–3-finger pinch, as well as tripod grip and lateral tripod grip.

To optimize the design, we defined the required functional glove workspace. We selected ten commonly used grasping postures in ADLs, including the previously mentioned five postures: medium wrap, thumb–2-finger pinch, lateral pinch, tripod grip, and power sphere, and five additional postures: precision disk, lateral tripod, thumb–3-finger, light tool, and index finger extension [18,19,20]. These postures can be represented by the joint angles of the index finger while performing the corresponding grasp [20,21,22,23,24,25,26,27]. A human finger movement can be characterized by a minimum of 2 DoFs [28,29,30]. Notably, the MCP and PIP joints exhibit significant influence on the workspace. Furthermore, there exists a correlated movement between the DIP and PIP joints [31]. Hence, we regarded the MCP and PIP joint angles as key features that defined the functional workspace. This assumption could express 84% of the fingertip workspace [32]. Table 1 shows the necessary MCP and PIP joint angle configurations of the index finger for performing the ten grasping postures. A full extension was defined as 0 deg, corresponding to the flexion angle. With these configurations, we defined a functional workspace for ten grasp postures in terms of the index finger joint angles (Figure 1). An area that included the joint angle configurations for the grasping postures was defined as the functional workspace for various grasping postures.

### 2.1. Finger–Robot Model

To investigate the workspace of the KADEX assisting the finger, we developed a comprehensive finger–robot model comprising a finger, cables, and a silicone strap. Although the model represents only the index finger, the same model can be generalized to the other fingers.

The finger model has three joints: MCP, PIP, and distal interphalangeal (DIP). Each joint is characterized by inherent passive stiffness and a neutral angle [33]. Consequently, we modeled each joint as a torsional spring with its corresponding stiffness and neutral angle. These joints can rotate from full extension (0 deg) to flexion (90 deg for the MCP and DIP joints, 120 deg for the PIP joint), covering the entire functional workspace depicted in Figure 1. For simplicity, we assumed a coupled relation between the DIP and PIP joint angles by introducing a proportional coefficient of 2/3 into the model [31].

The silicone strap, which is routed along the dorsal side of the finger, imparts tension for finger extension. The stiffness of the strap ($k_{ext}$) was determined by considering Young’s modulus of the silicone material (DragonSkin20, Smooth-On. Inc., Macungie, PA, USA) and the geometric attributes of the strap. The strap undergoes elongation during joint flexion because of its location on the dorsal side of the joint. As a result, the strap generates an extensional torque relative to the joint angles. Additionally, the strap is prestretched to a length of $x_{prestretch}$ to consistently provide extension torque, facilitating joint extension and compensating for the neutral angle.

**Figure 2:**
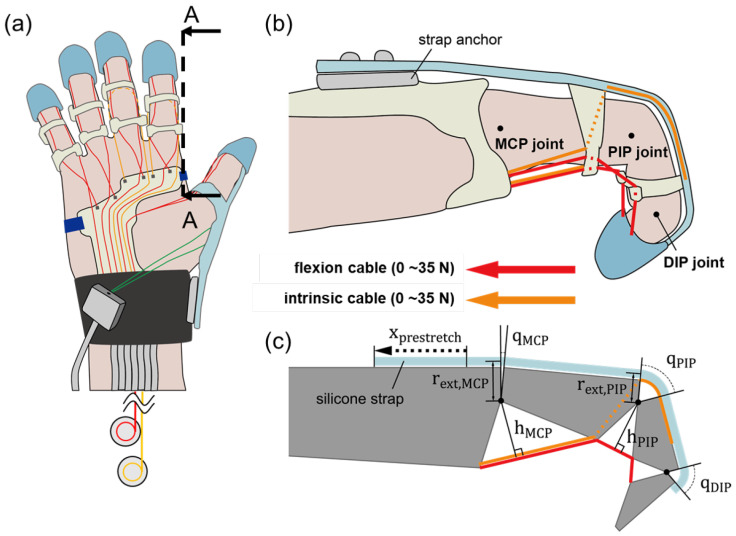
(**a**) Routing design of KADEX. Red and orange lines represent the flexion and intrinsic cable, respectively. (**b**) Intersectional view of A–A. Cable routing and silicone strap structure of the soft robotic glove (index finger). (**c**) Schematic of the finger–robot model.

Given the two cable routings designed to transmit torque to the joints, each routing is characterized by its anchoring position (Figure 2). The flexion cable follows the palmar side of the finger (depicted by the red line in Figure 2), anchoring to each phalanx and transmitting flexion torque to the joints. In contrast, the intrinsic cable is routed from the dorsal side of the PIP joint, passing through the space beside the proximal phalanx and toward the palm (as indicated by the orange line in Figure 2). It then transmits flexion torque to the MCP joint and extension torque to the PIP joint. Each cable was modeled to be tensioned up to 35 N [17]. The dimension parameters presented in Figure 2c were measured after the subject wore the glove due to variations in hand sizes. Equations (1) and (2) can be used to derive the net joint torques on the MCP and PIP joints, respectively. The robot’s workspace was calculated by establishing equilibrium conditions for $q_{MCP}$ and $q_{PIP}$ using two equations, varying the cable tension from 0 to 35 N. As a result, velocity-dependent terms, such as damping and inertia, were not considered in this analysis.

(1) $$\begin{array}{c} MCPjointnettorque=-k_{MM}*q_{MCP}-k_{MP}*q_{PIP}+k_{MCP}*q_{MCP,neutral}+\\ \left(T_{flexion}+T_{intrinsic}\right)*h_{MCP}-k_{ext}*x_{prestretch}*r_{ext,MCP} \end{array}$$

(2) $$\begin{array}{c} PIPjointnettorque=-k_{MP}*q_{MCP}-k_{PP}*q_{PIP}+k_{PIP}*q_{PIP,neutral}+\\ T_{flexion}*h_{PIP}-T_{intrinsic}*r_{ext,PIP}-k_{ext}*x_{prestretch}*r_{ext,PIP}. \end{array}$$

where the effective joint stiffness $k_{MM}$, $k_{PP}$, $k_{MP}$, and $k_{PM}$ are given in (3)–(6):

(3) $$k_{MM}=k_{MCP}+k_{ext}*r_{ext,MCP}^{2}$$

(4) $$k_{PP}=k_{PIP}+k_{ext}*r_{ext,MCP}*\left(r_{ext,PIP}+2/3*r_{ext,PIP}\right)$$

(5) $$k_{MP}=k_{ext}*r_{ext,MCP}*r_{ext,MCP}$$

(6) $$k_{PM}=k_{ext}*r_{ext,MCP}*\left(r_{ext,MCP}+2/3*r_{ext,PIP}\right).$$

Physiological parameters, such as joint stiffness and neutral angles, were obtained from a patient with chronic stroke who exhibited moderate spasticity (Modified Ashworth Scale (MAS) grade 1) in the right hand, with joint stiffness surpassing that of a healthy individual. The experimental protocols were approved by the Institutional Review Board at the Korea Advanced Institute of Science and Technology (KH2018-111); written informed consent was obtained from the subjects before participation. Neutral angles were measured while the subjects relaxed their hands. The joint stiffness was then calculated by dividing the torque required for full extension by the neutral angle based on the linear assumption of stiffness [33]. The neutral angle and joint stiffness of the MCP joint are denoted as $q_{MCP,neutral}$ and $k_{MCP}$, respectively. Similarly, the parameters for the PIP joint follow the same naming convention but with the PIP notation.

**Figure 3:**
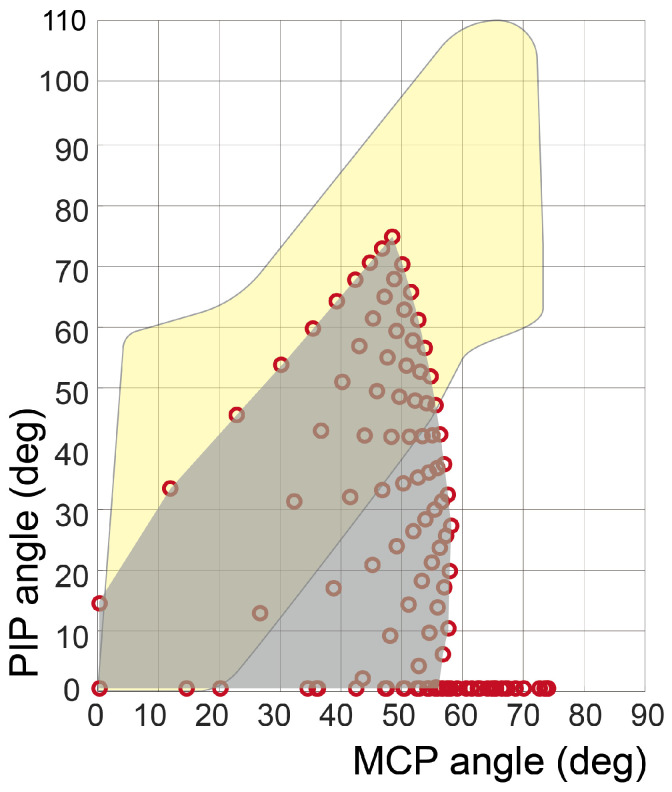
Workspace simulation from the stroke subject’s finger–robot model. The red dots represent the numerical calculation of Equations (1) and (2), while $T_{flexion}$ and $T_{intrinsic}$ varied from 0 to 35 N. The gray-shaded region represents the robot’s workspace, which is an interpolation of red dots. The yellow-shaded region represents the functional workspace for various grasping tasks obtained from Figure 1.

We conducted simulations to determine the equilibrium angles of the joints based on varying cable tensions. As the equations above are expressed in implicit functions with respect to the joint angles (with the distance h being a function of the joint angles), we used MATLAB R2021a to calculate the equalized joint angles numerically. Figure 3 compares the simulated workspace of the glove (gray area) and the functional workspace (yellow area).

### 2.2. Workspace Analysis and Design Parameter Optimization

**Figure 4:**
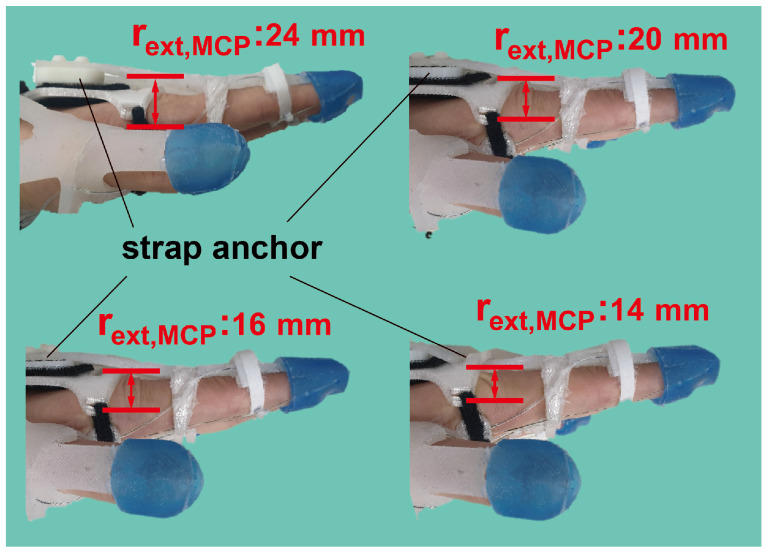
$r_{ext,MCP}$ can be adjusted by altering the thickness of the strap anchor on the back of the hand. It was adjusted from 24 to 14 mm.

The simulation results presented in Figure 3 show that the initial design was insufficient to assist in postures involving precision disk and power sphere grasping. These postures necessitate the PIP joint flexion to 50 degrees while maintaining the MCP joint extended at 0 degrees. However, as the cable tension increases, the MCP joint tends to flex before the PIP joint angle reaches 50 degrees, as the extension is assisted by the underactuated mechanism. To enhance the extensional torque applied to maintain the MCP joint extension, the moment arm of the silicone strap ($r_{ext,MCP}$) needed elongation. Nevertheless, excessive elongation of the moment arm may interfere with MCP joint flexion in other grasping postures. Therefore, the minimum moment arm length required to facilitate precision disk grasping was determined from Equations (1) and (2), assuming $q_{MCP}$ and $q_{PIP}$ were 0 and 50 degrees, respectively. To sustain this posture, the net torque of each joint should be equal to zero. Consequently, the minimum required length of $r_{ext,MCP}$ can be derived using Equations (7) and (8). Notably, there are two variables ($T_{flexion}$ and $r_{ext,MCP}$) and the others are all known, and thus, the equations are solvable. The derived $r_{ext,MCP}$ can be easily adjusted by altering the thickness of the strap anchor on the back of the hand (Figure 4).

(7) $$\begin{array}{c} MCPjointnettorque=-k_{MP}*\left(50deg\right)+k_{MCP}*q_{MCP,neutral}+\\ T_{flexion}*h_{MCP}-k_{ext}*x_{prestretch}*r_{ext,MCP}=0 \end{array}$$

(8) $$\begin{array}{c} PIPjointnettorque=-k_{PP}*\left(50deg\right)+k_{PIP}*q_{PIP,neutral}+\\ T_{flexion}*h_{PIP}-k_{ext}*x_{prestretch}*r_{ext,PIP}=0. \end{array}$$

**Figure 5:**
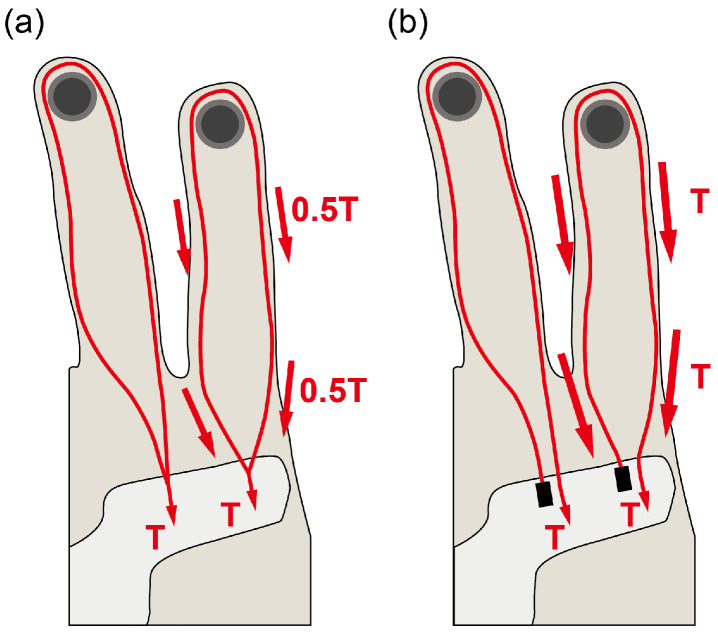
(**a**) Pulling both ends of the cable and (**b**) the new design anchors one end of the cable to the palmar structure and pulls the other end. Anchoring to the palmar structure is shown as a black rectangle.

The optimal moment arm length was identified to attain a workspace suitable for precision disk and power sphere grasping. The cable tensions required to assist with the ten grasping postures were calculated using the finger–robot model and are presented in Table 2. The maximum tension required for the flexion and intrinsic cables amounted to 70.2 and 20.4 N, respectively, exceeding the maximum tension capacity (35 N) that the servomotors (DCX 32, 70 W, maxon motor AG) can provide [17]. To address this limitation, a novel routing design was implemented to amplify the torque applied to the joints. Instead of pulling both cable ends (Figure 5a), the new design anchors one end of the cable to the palmar structure and pulls the other end (Figure 5b). This routing approach effectively doubles the torque exerted on the joints by employing the principle of a moving pulley. Consequently, maintaining the same posture requires twice the stroke length but only half the tension.

**Figure 6:**
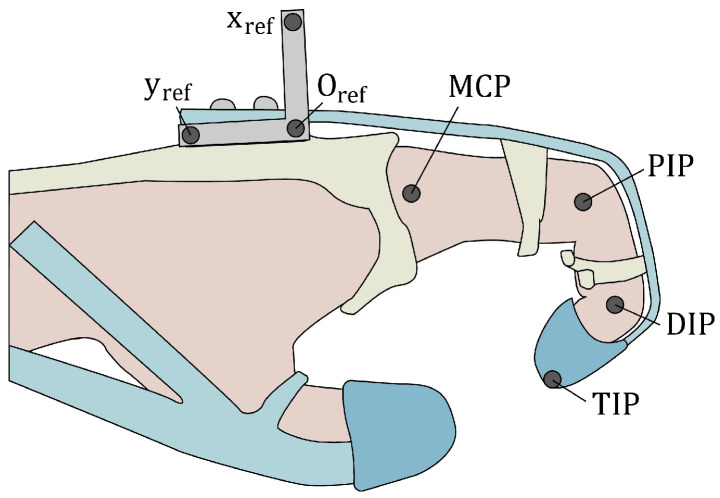
Positions of the reflective markers for motion capture. The MCP, PIP, DIP, and TIP markers were projected onto the reference plane ($x_{ref}$, $y_{ref}$, $O_{ref}$) to calculate the joint angles.

Following the optimization of the design, which included the moment arm length and routing design, we evaluated the workspace of the stroke subjects. Joint trajectories were measured using a motion-capturing system (V120:Trio, NaturalPoint, Inc., Corvallis, OR, USA). Seven reflective markers were attached to the hand to capture the joint angles (Figure 6). The cables were individually tensioned using electrical servomotors (DCX 32, 70W, maxon motor AG, Sachseln, Switzerland), with each one equipped with a pulley measuring 8 mm in diameter. The tension exerted on the cables was transferred via a Bowden cable to position the actuator system remotely from the glove, as illustrated in Figure 2a. Control over the system was achieved using a motor driver (ESCON50/5, maxon motor AG, Sachseln, Switzerland) operated through LabVIEW (LabVIEW 2016, National Instruments, Austin, TX, USA). The cable tensions were applied in the range of 0–35 N in the following sequence: release (both cables had 0 N tension), flexion (35 N), both flexion and intrinsic, intrinsic, and release. This sequence was repeated thrice. Throughout the evaluation, the subjects were instructed to relax while the robot assisted their fingers, mitigating the impact of volitional movements. Tension was promptly released if the subject experienced any pain or discomfort.

To evaluate the performance of the optimized design in providing assistance, we measured the pinching force and elapsed time required to assist with a posture while the device was in operation. To quantify the pinching force, we instructed the subject to perform the thumb–2-finger pinch while the device was actively assisting. The device applied the corresponding torque for the thumb–2-finger pinch, which was calculated using Equations (7) and (8). A force sensor (Nano17, ATI Industrial Automation, Apex, NC, USA) was positioned between the thumb and the other two fingers (index and middle fingers) to measure the pinching force. During pinching, the force sensor was placed between the tips of the thumb and other two fingers, and a thumb–2-finger pinch with a width of 2 cm was executed. The elapsed times for pinching and releasing (returning to the extension posture) were defined as the settling time of the movement, representing the time taken for 95% of the final joint angle to be reached while the device was assisting the thumb–2-finger pinch. The same motion capture protocol employed the same marker positions as those depicted in Figure 6. In addition, the reference torque trajectory of the motor was set to linearly rise and fall within 0.5 s.

## 3. Results

**Figure 7:**
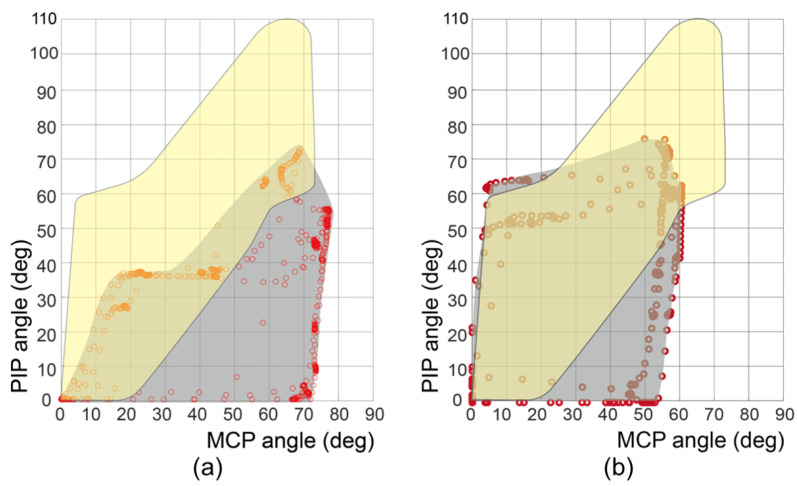
Motion-captured workspace of a stroke subject’s index finger with (**a**) previous design ($r_{ext,MCP}$ = 14 mm, previous cable routing) and (**b**) optimized design ($r_{ext,MCP}$ = 24 mm, torque-amplified cable routing). The red dots represent the measured joint angle configurations. The gray-shaded region represents the glove’s assistive workspace. The yellow-shaded region represents the functional workspace for various types of grasping.

Figure 7 and supplementary Video S1 shows the motion-captured workspace of a stroke subject with a previous and optimized design, highlighting the substantial difference achieved by the routing design. In the prior design, the MCP joint extension moment arm ($r_{ext,MCP}$) measured 14 mm, whereas in the optimized version, this length was increased to 24 mm. The workspace of the previous design covered only the lower-right portion of the functional workspace. Thus, the previous design can assist seven postures, except for the power sphere, precision disk, and light tool grasp. However, the workspace of the optimized design covered more than half of the functional workspace. The area where the optimized design could assist contained nine grasping postures, except for the light tool grasp.

The robot-assisted pinching force amounted to 10.4 N, including the volitional pinching force exerted by the subject. The elapsed times to achieve and release the pinching posture, reaching 95% of the final joint angle, were 0.75 s and 0.46 s, respectively.

## 4. Discussion

The comparison in Figure 7 demonstrates the notable difference in the workspace between the previous and optimized designs. The application of the optimal design parameter, specifically $r_{ext,MCP}$, was effective in facilitating grasping postures for large objects (such as a precision disk and power sphere), which corresponded to the upper-left region of the functional workspace. The introduction of the moving pulley routing mechanism also enabled postures suitable for grasping small objects (e.g., lateral pinch and thumb–two-finger pinch), involving simultaneous flexion of the PIP and MCP joints, with torque amplification to the joint. The experimental results confirmed that the optimized design parameters provided the robot with sufficient workspace assistance for nine grasping postures out of ten. We anticipate that augmenting the tension of the flexion cable, as outlined in Table 2, will enable the attainment of the requisite workspace for accommodating the posture required for the light tool.

The functional workspace defined in this paper includes the standard deviation of joint angles to accommodate intersubject variation in grasp postures. Moreover, the finger–robot model can be generalized to different subjects if their finger characteristics are quantified.

This study established the foundation for optimizing the design of soft gloves that employ underactuated mechanisms. However, the practicality of measuring individual joint stiffness values and computing the optimal moment arm length for clinical or daily use may be limited. As an alternative, we propose adjusting the moment arm based on hand posture. For instance, if the glove encounters difficulty in assisting with grasping large objects due to premature flexion of the MCP joint, increasing the moment arm of MCP extension could prove beneficial. Conversely, if the glove struggles with small pinching postures, reducing the moment arm of MCP extension and increasing the flexion moment arm may be more effective.

In this study, we introduced a method to easily adjust the moment arm length by altering the thickness of the routing anchor. This approach presents a practical and adaptable solution for modifying the moment arm length based on observed limitations in hand postures during glove usage.

Moreover, when enlarging the moment arm on the palmar side of the finger is not desirable because of interference with grasping, an alternative approach can reinforce the flexion mechanism. In this study, we employed a moving pulley mechanism to enhance the flexion torque on the joint to avoid adding bulk to the actuating module. However, there are alternative options, such as increasing the motor power or incorporating a gear system into the motor, which can also achieve the desired reinforcement of the flexion mechanism without compromising the compactness of the glove design.

The robot’s performance was also evaluated in terms of the speed and force it can provide during assistance. According to previous studies, a comfortable assisting time for a posture should generally fall within the range of 1 to 2 s [34]. It is worth noting that individuals with neurological diseases are often characterized by spasticity (reflex muscle tone response to rapid movement); therefore, the speed of the robot should be limited to prevent injury [35,36]. In this study, the finger joint angle speed was consistently maintained below 200 deg/s to ensure the minimum operating time for the robot was approximately 0.5 s in the absence of any resistance [37]. Even when considering the subject’s finger with its inherent stiffness, the glove successfully assisted in grasping and releasing the finger within 0.75 and 0.46 s, respectively. These results demonstrate the robot’s capability to achieve comfortable and safe assisting times, which is particularly important in the context of individuals with neurological conditions.

The robot’s assisting pinching force, which was measured at 10.4 N, is crucial for aiding ADL tasks, such as zipping, using keys, or holding food utensils [38]. Therefore, the robot can provide sufficient pinching force for various ADL-related rehabilitation tasks. However, it is essential to recognize that the maximum grasping force is constrained by the soft material of the robot. According to previous studies, most soft robots designed for hand rehabilitation typically offer maximum assistance of around 15 N, whereas exoskeleton robots can provide more than 30 N of force [13,39,40,41]. Although a grasping force of 10 N is adequate for tasks that involve lightweight objects, it may not be sufficient for more demanding activities, such as inserting and unplugging electric plugs, which highlights the limitations of soft robots for broad applications [38].

## 5. Conclusions

We introduced a finger–robot model designed to simulate the workspace of a cable-driven underactuated soft robotic glove. Through the optimization of the design parameters, we successfully facilitated the functional workspace of various grasping postures. The identified optimal design parameters were instrumental in expanding the glove’s workspace, which is a crucial consideration before the fabrication and utilization of the glove. Additionally, the evaluation of the grasping speed and force ensured the glove’s usability across a spectrum of rehabilitation tasks.

After the design optimization, the glove could assist a larger area of the functional workspace. Although the target functional workspace could vary according to the purpose of the glove, a similar approach can be applied to optimize the design parameters. Also, the finger–robot model can be advanced, such as by adding the other DoF (DIP joint flexion/extension, finger abduction/adduction) or applying the other glove design. For example, this approach can be a useful tool for designing a single-DoF soft robotic glove. In this case, optimization can adjust the moving sequence of the MCP and PIP joints. Although the final posture is identical, the trajectory to reach that posture may be more natural. Thus, it can provide ergonomic assistance to users.

## Figures and Tables

**Table 1:** Index finger joint angles for ten grasping postures.

Grasping Type	MCP Joint Angle ± SD (deg)	PIP Joint Angle ± SD (deg)
Medium wrap [21]	63 ± 11.6	66 ± 8.85
Thumb–2-finger pinch [22]	58 ± 7	76 ± 13
Thumb–3-finger pinch [22]	58 ± 7	76 ± 13
Lateral pinch [22]	62 ± 8	76 ± 8
Tripod grip [23]	48 ± 7.8	53 ± 10.2
Lateral tripod [23]	48 ± 7.8	53 ± 10.2
Power sphere [24]	21.9	63.7
Precision disk [25]	7.26 ± 3.29	56.2 ± 3.09
Light tool [26]	65.6 ± 7.9	105.5 ± 9.2
Index finger extension [27]	0	0

**Table 2:** Required cable tensions for ten grasping postures.

Grasping Type	Flexion Cable Tension (N)	Intrinsic Cable Tension (N)
Medium wrap	39.3	20.4
Thumb–2-finger pinch	36.9	10.5
Thumb–3-finger pinch	36.9	10.5
Lateral pinch	40.5	14.4
Tripod grip	22.2	11.4
Lateral tripod	22.2	11.4
Power sphere	14.9	2.9
Precision disk	10.5	0
Light tool	70.2	0.97
Index finger extension	0	0

## Data Availability

Data are contained within the article and Supplementary Materials.

## Supplementary Materials

The following supporting information can be downloaded from https://www.mdpi.com/article/10.3390/biomimetics9030172/s1—Video S1: Workspace comparison previous design versus optimized design.

## Abbreviations

The following abbreviations are used in this manuscript:

ADL	Activity of daily living
SCI	Spinal cord injury
KADEX	KAIST dexterous glove
DoF	Degree of freedom
MCP	Metacarpophalangeal
PIP	Proximal interphalangeal
DIP	Distal interphalangeal
MAS	Modified Ashworth Scale
